# Combined Low Dose of Ketamine and Social Isolation: A Possible Model of Induced Chronic Schizophrenia-Like Symptoms

**DOI:** 10.1192/j.eurpsy.2022.505

**Published:** 2022-09-01

**Authors:** D. Khalifa, L. Rashed, A. Shamseldeen, S. Stephan

**Affiliations:** 1 Kasr Al Ainy hospitals, Cairo University, Psychiatry, Cairo, Egypt; 2 Kasr Al Ainy hospitals, Cairo University, Biochemistry, Cairo, Egypt; 3 Kasr Al Ainy hospitals, Cairo University, Physilogy, Cairo, Egypt

**Keywords:** Model, Ketamine, schizophrénia

## Abstract

**Introduction:**

Identifying a feasible model of chronic schizophrenia would be valuable for studying the possible underlying mechanism and to investigate emerging treatments. Our hypothesis starts from the observation that combining ketamine with isolation could result in long-lasting neuro-psychological deficits and schizophrenia-like features; thus, it could probably be used as the first model of chronic schizophrenia that emphasizes the characteristic of having a multifactorial etiology

**Objectives:**

creation of a complex animal model capable of exhibiting the multifactorial origin and manifestation of schizophrenia.

**Methods:**

we investigated the effects of ketamine administration combined with isolation in inducing schizophrenia-like symptoms in male albino rats and the brain reactive oxygen species levels.

**Results:**

Our results showed that the number of lines crossings in the open field test, the number of open arm entries in the elevated plus maze, and the spontaneous alternations percentage in the Y-maze were significantly lower in the ketamine + isolation group compared to both the control and ketamine + social housing group (p < 0.05). Furthermore, the ketamine + isolation intervention significantly increased the MDA levels and decreased the GPx levels both in the hippocampus and the cortex of the rats. In addition, our premise of creating a model capable of exhibiting both positive and negative symptoms of schizophrenia was also based on adding the aripiprazole treatment to a group of rats

**Conclusions:**

combining ketamine with isolation could result in long-lasting neuro-psychological deficits and schizophrenia-like features

**Disclosure:**

No significant relationships.

